# Diagnostic stewardship in infectious diseases: a scoping review

**DOI:** 10.1099/jmm.0.001831

**Published:** 2024-05-09

**Authors:** Robert Shorten, Kate Pickering, Callum Goolden, Catherine Harris, Andrew Clegg, Hill J

**Affiliations:** 1 Department of Microbiology, Lancashire Teaching Hospitals NHS Foundation Trust, Foundation Trust, UK; 2 The University of Manchester, Manchester, UK; 3 University of Central Lancashire, Fylde Rd, Preston PR1 2HE, UK

**Keywords:** antimicrobial stewardship, diagnostic stewardship, infectious diseases, review

## Abstract

**Introduction.** The term ‘diagnostic stewardship’ is relatively new, with a recent surge in its use within the literature. Despite its increasing popularity, a precise definition remains elusive. Various attempts have been made to define it, with some viewing it as an integral part of antimicrobial stewardship. The World Health Organization offers a broad definition, emphasizing the importance of timely, accurate diagnostics. However, inconsistencies in the use of this term still persist, necessitating further clarification.

**Gap Statement.** There are currently inconsistencies in the definition of diagnostic stewardship used within the academic literature.

**Aim.** This scoping review aims to categorize the use of diagnostic stewardship approaches and define this approach by identifying common characteristics and factors of its use within the literature.

**Methodology.** This scoping review undertook a multi-database search from date of inception until October 2022. Any observational or experimental study where the authors define the intervention to be diagnostic stewardship from any clinical area was included. Screening of all papers was undertaken by a single reviewer with 10% verification by a second reviewer. Data extraction was undertaken by a single reviewer using a pre-piloted form. Given the wide variation in study design and intervention outcomes, a narrative synthesis approach was applied. Studies were clustered around common diagnostic stewardship interventions where appropriate.

**Results.** After duplicate removal, a total of 1310 citations were identified, of which, after full-paper screening, 105 studies were included in this scoping review. The classification of an intervention as taking a diagnostic stewardship approach is a relatively recent development, with the first publication in this field dating back to 2017. The majority of research in this area has been conducted within the USA, with very few studies undertaken outside this region. Visual inspection of the citation map reveals that the current evidence base is interconnected, with frequent references to each other’s work. The interventions commonly adopt a restrictive approach, utilizing hard and soft stops within the pre-analytical phase to restrict access to testing. Upon closer examination of the outcomes, it becomes evident that there is a predominant focus on reducing the number of tests rather than enhancing the current test protocol. This is further reflected in the limited number of studies that report on test performance (including protocol improvements, specificity and sensitivity).

**Conclusion.** Diagnostic stewardship seems to have deviated from its intended course, morphing into a rather rudimentary instrument wielded not to enhance but to constrict the scope of testing. Despite the World Health Organization’s advocacy for an ideology that promotes a more comprehensive approach to quality improvement, it may be more appropriate to consider alternative regional narratives when categorizing these types of quality improvement interventions.

## Data Summary

The data that were used within this paper are available on request from James Hill Email: Jehill1@uclan.ac.uk

## Introduction

The growing challenges facing healthcare systems appear unrelenting [1,2]. Ageing populations, increase in chronic conditions, staffing shortages and the effects of the era of living with COVID-19 place health services under pressure [3,5]. With demand for healthcare anticipated to rise globally during the ensuing decade, there is an increasing emphasis on using constrained resources appropriately [6,7]. Given that diagnostic services are a fundamental part of care pathways, they are not immune to these pressures, and it is important that they operate effectively and efficiently [3,8, 9]. Although a relatively new term [10], diagnostic stewardship has received increased attention during the last 10 years as concern has grown around the appropriate use of resources to deliver high-quality effective healthcare [9]. Despite the increased use of the term, its precise definition is yet to be established, which is important to ensure appropriate use of the strategy in managing care [11].

‘Antimicrobial stewardship’ is defined by the National Institute for Health and Care Excellence as ‘an organisational or healthcare-system-wide approach to promoting and monitoring judicious use of antimicrobials to preserve their future effectiveness’ [12]. This approach is designed to prevent inappropriate antimicrobial prescribing, which can cause direct patient harm in the form of adverse effects including healthcare-associated infections, and the development of antimicrobial resistance (AMR) [13]. The essence of antimicrobial stewardship programmes is to ensure that the right patient receives the right antimicrobial, at the right time, via the right route, at the right dose and for the right duration [14]. The logical extension should suggest that ‘diagnostic stewardship’ is performing the right test, on the right patient, at the right time. Editorials and reviews have attempted to define the term [9,15,17], and some have detailed the various stages where interventions may be applied in the pre-analytical, analytical and post-analytical phases [9]. However, publications, including systematic reviews [18], retrospective studies [19,20] and multi-centre validation of machine learning tools [21], have used the term without defining it. One collaborative study regarding paediatric blood cultures (BCs) defined diagnostic stewardship as “optimising the use of diagnostic tests to improve treatment decisions” [22]. Conversely, members of two subgroups of the European Society for Clinical Microbiology and Infectious Diseases have expressed concerns about the use of the term [11]. Diagnostic stewardship should not be a standalone practice but rather an integral part of antimicrobial stewardship [11]. ‘Diagnostic test appropriateness’ was proposed as an alternative term to engage with non-diagnostic specialists, although the definition of the former term has not yet been formalized [11].

As part of its Global Antimicrobial Resistance Surveillance System (GLASS), the World Health Organization (WHO) has defined diagnostic stewardship as ‘coordinated guidance and interventions to improve appropriate use of microbiological diagnostics to guide therapeutic decisions . It should promote appropriate, timely diagnostic testing, including specimen collection, and pathogen identification and accurate, timely reporting of results to guide patient treatment’ [23]. In this context, GLASS aims to deliver ‘patient management guided by timely microbiological data to deliver safer and more effective and efficient patient care’, and ‘accurate and representative AMR surveillance data to inform treatment guidelines, and AMR control strategies’ [23]. This WHO initiative is targeted to improve access to standardized diagnostics at AMR surveillance sites, particularly in low- and middle-income countries. Thus, it is evident that there are notable inconsistencies regarding the use of diagnostic stewardship as a term, and further clarification of the nomenclature is required. We endeavour to undertake this task by categorizing the present application of the term within the current published literature.

## Aims

The primary aim of this scoping review is to categorize the use of diagnostic stewardship interventions within the current literature. The secondary aims are to define diagnostic stewardship by identifying common characteristics and factors of its use within the literature.

## Methods

Prior to commencement, this scoping review was pre-registered on the open science framework registry (see https://doi.org/10.17605/OSF.IO/WYEKB). The scoping review methodology followed the guidance provided by Peters et al. (2015) and Levac et al. (2010)] [24,25].

### Search

The following electronic bibliographic databases were searched for studies where an intervention was defined as diagnostic stewardship: MEDLINE (Ovid), PubMed (https://pubmed.ncbi.nlm.nih.gov/advanced/), Embase (Ovid), The Cochrane Library (all databases via Wiley), CINAHL (EBSCOhost), Web of Science, ClinicalTrials.gov registry and WHO International Clinical Trials Registry Platform from date of inception to October 2022 (see supplement file 1, available in the online version of this article for MEDLINE search strategy example). No language or other limits were applied to the searches. Furthermore, an additional search was conducted using Google Scholar via Harzing’s Publish or Perish (Windows GUI Edition 8.2.3944.8118) leading to the retrieval of a software restricted maximum of 1000 results. The identification of duplicate entries was initially carried out with EndNote and subsequently cross-checked with Rayyan, with verification performed by a single reviewer (J.H.).

### Study selection

Any type of observational or experimental study (including conference abstracts) where the author explicitly defined the intervention as ‘diagnostic stewardship’ from any clinical area (primary, secondary and tertiary care settings) was included. As the aim was to include as many studies as possible, there was no specified outcome set.

Abstract and title screening was carried out by a single reviewer using Rayyan. This selection process was piloted with 10% of retrieved abstracts and titles being screened by a second reviewer independently and resolved before undertaking the remaining screening [26]. A Kappa Score was calculated for this piloted screening process, and substantial agreement (0.61–0.80) was required before continuation. If this could not be achieved, increments of 10 % were screened until substantial agreement could be achieved. Full-paper screening used the same 10 % verification process, and reasons for exclusion were recorded and reported during full-paper screening.

## Data extraction (selection and coding)

Data extraction was undertaken by a single reviewer using a pre-piloted form. The data items extracted included the country of study, town/city, study type, clinical setting, single or multi-centre, population, sample size, diagnostic focus, specimen type, test type, pathology discipline and outcomes.

## Strategy for data synthesis

Due to the expected wide variation in study design, interventions and outcomes, a narrative synthesis approach was used to structure the findings. Studies were clustered around common diagnostic stewardship interventions where appropriate. A citation count per year was presented using bar charts to highlight the field’s growth within this area over time. A geographical location map of the study location was used to identify any commonality regarding areas of use of diagnostic stewardship [27]. A citation map was generated using Litmaps citation mapping software to assess the inter-relationship of citations within this field and help review the success of forward and backward citation searching within this area of study [28]. To visualize the type of intervention/stage of process of interventions defined as diagnostic stewardship, a visual representation of number of studies per stage was generated.

## Results

A total of 2556 records were identified from the initial search. After duplicate removal, a total of 1310 citations were identified, with 237 articles retrieved after title and abstract screening and 105 studies (*n* = 109 citations) included in the scoping review following full-paper screening. For the 10 % verification process, substantial agreement was achieved between all three reviewers (percentage of agreement: 86.23–88.67, Cohen’s *k*: 0.62–0.69).

**Fig. 1.**
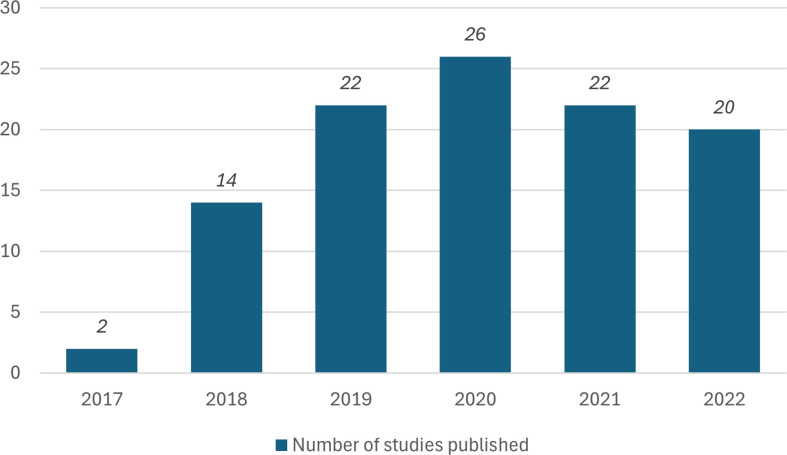
Number of publications per year.

The first study that classified their intervention to be a diagnostic stewardship approach was published in 2017 (see Fig. 1 for number of publications per year and Table 1 for full study characteristics). Subsequently, there has been a consistent increase in intervention studies classified as employing a diagnostic stewardship approach, with 26 studies recorded in the year 2020, and from this period, there has been ≥20 studies published each year.

**Table 1.** Study characteristics

Author	Year	Country	Study type	Sample size at start of study	Clinical setting coded (RS)	Specimen type	Diagnostic focus	Intervention	Stage of process	Outcomes
**Abbas *et al.***[36]	2021	Switzerland	Retrospective before and after	Not recorded	Tertiary care	Blood	Procalcitonin	Restricted access (hard stop)	Pre-analytical	The primary outcome was the change in antibiotic use in defined daily doses per 1000 PDs per month. The secondary outcomes included DOT per 1000 PDs per month, in-hospital mortality, LOS and cost savings
**Abbasi *et al.*** [37]	2020	USA	Retrospective before and after	Not recorded	Tertiary care	Stool	*Clostridiumdifficile*	Restricted access (hard stop)	Pre-analytical	HO-CDI rate per 10 000 PDs and testing volume
**Acuna *et al.*** [38]	2022	Chile	Prospectively before and after	409 Cerebrospinal fluid (CSF) samples Fig. 5 were analysed, 297 pre-intervention and 112 post-intervention	Inpatients (level of care not specified)	CSF	Central nervous system (CNS) infection	Access to an improved diagnostic test	Analytical	Median intensive care units (ICU) days, ICU bed day cost, comparison of aetiological infectious agents identified between pre- and post-intervention groups
**Agarwal *et al.*** [39]	2021	India	Retrospective before and after	486 BCs positive for bloodstream infections (BSI) from ICU	Tertiary care	Blood	BCs	Access to an improved diagnostic test	Analytical	Total number of blood samples received from patients with suspected BSI from ICU, the number of samples that tested positive for BSI, microbiological aetiology of BSI, antimicrobial susceptibility of bacteria between pre- and post-intervention, antimicrobial usage
**Al-Bizri *et al.*** [40]	2022	USA	Prospectively before and after	21 367 Patients admitted to the ICU	Tertiary care	Urine	Catheter-Associated Urinary Tract (CA-UTI	Education	Pre-analytical	Were also evaluated for 30-day re-admission for using
**AlQahta *et al.*[Bibr R40]** [41]	2021	USA	Before and after	65 Adult patients who had positive BCs with Gram-positive cocci in clusters	Tertiary care	Blood	BCs	Access to an improved diagnostic test	Analytical	Time to optimal therapy in hours, number of patients switched to targeted therapy, number of patients targeting higher vancomycin trough goal for methicillin-resistant S. aureus bacteraemia, duration of inpatient antibiotic therapy, length of hospital stay, 30-day mortality rate, 30-day relapse rate
**Andrade *et al.*** [42]	2019	USA	Prospectively before and after	Not recorded	Not recorded	Blood	Hepatitis C virus (HCV)	Automated reflex testing	Post-analytical	Number of reactive HCV Ab tests with subsequent hepatitis C virus ribonucleic acid viral load testing performed, number of positive HCV Ab tests with previous positive Ab results.
**Baghdadi[Bibr R42]** [43]	2020	USA	Prospectively before and after	Not recorded	Inpatients (level of care not specified)	Stool	*C.difficile*	Restricted access (soft stop) and laboratory rejection of inappropriate samples and education of requesters and automated reflex testing	Pre-analytical and post-analytical.	HO-CDI cases per 10 000 acute care bed days, isolation days per 10 000 acute care bed days, doses of oral vancomycin per 10 000 acute care bed days and complications per 10 000 acute care bed days
**Beaver[Bibr R43]** [44]	2021	USA	Retrospective before and after	1210 Charts screened	Not recorded	CSF	CNS infection	Automated reflex testing	Post-analytical	CSF white Blood Cells count, organism identified by PCR panel, sensitivity, specificity, positive predictive value, negative predictive value and Utilization Reduction for three different reflex approaches
**Bettger[Bibr R44]** [45]	2022	USA	Retrospective before and after	216 PCR tests	Army medical facility	Stool	*C.difficile*	Access to an improved diagnostic test. Automated reflex testing	Analytical and post-analytical	Number of patients treated for *Clostridium difficile* infection (CDI), median LOS (days), mortality rate at 30 days, readmission for CDI at 30 days
**Bianchini** [46]	2019	USA	Prospectively before and after	91 Patients pre-intervention and 91 patients post-intervention	Tertiary care	Multiple	Respiratory pathogens	Restricted access (soft stop)	Pre-analytical	Primary outcome: duration of antimicrobial therapy. Secondary outcomes: diagnostic tests, antimicrobial de-escalation, LOS, mortality, retreatment, 30-day readmission and *C. difficile* incidence
**Bilinskay** [47]	2018	USA	Prospectively before and after	483 Best practice alerts triggered on 366 patients	Secondary and tertiary care	Stool	*C.difficile*	Restricted access (soft stop)	Pre-analytical	Percent of accepted vs overridden alerts, percent of *C. difficile* tests ultimately ordered by providers for patients with triggered best-practice alerts (BPAs), percent of ordered tests successfully completed by the laboratory and the rates of four clinical signs and symptoms of CDI in the PCR-positive and PCR-negative groups
**Bischoff[Bibr R47]** [48]	2017	USA	Prospectively before and after	Not recorded	Tertiary care	Stool	*C.difficile*	Access to an improved diagnostic test. Automated reflex testing	Analytical and post-analytical	Rates of enterocolitis due to *C. difficile*, National Healthcare Safety Network (NHSN) *C. difficile* LabID reportable events, CDI complications, mortality, antimicrobial prescription patterns, cluster occurrences; testing, treatment and isolation costs
**Block[Bibr R48]** [49]	2018	USA	Time series	Not recorded	Inpatients (level of care not specified)	Stool	*C.difficile*	Restricted access (soft stop)	Pre-analytical	NHSN *C. difficile* hospital-onset standardized infection ratio, inpatient facility *C. difficile* healthcare facility-onset incidence rate per 10 000 PDs
**Bradsha[Bibr R49]** [50]	2021	USA	Before and after	99 Thyroid-stimulating hormone (TSH) orders pre-intervention, 99 TSH orders post-intervention	Tertiary care	Blood	Thyroid function tests	Restricted access (soft stop)	Pre-analytical	Number and percentage of inappropriate TSH tests ordered before and after implementing the three interventions, cost savings, inappropriate changes in thyroid therapy based on improperly ordered tests and the number of free T4 lab tests ordered on patients with a TSH within the therapeutic range
**Broadhu[Bibr R50]** [51]	2020	USA	Before and after	459 Meningitis/encephalitis panel tests ordered for 263 patients	Tertiary care	CSF	CNS infection	Automated reflex testing	Post-analytical	ME panel test utilization rate, negative predictive value of non-pleocytic CSF samples, test yield and false-positivity rate, and time to appropriate de-escalation of acyclovir
**Broyles *et al.***[52]	2021	USA	Before and after	1303 Urine samples	Emergency department	Urine	Urinary tract infection (UTI)	Education	Pre-analytical	Specimen contamination rate for urinalysis (epithelial cell count ≥5 cells/high power field) and urine culture procedures
**Christensen *et al.*[Bibr R52]** [53]	2019	USA	Quasi-experimental	743 CDI PCR results	Tertiary care	Stool	*C.difficile*	Restricted access (soft stop)	Pre-analytical	Incident rate of HO-CDI per 10 000 PDs, monthly CDI standardized infection ratio and consumption of oral vancomycin DOT per 1000 PDs
**Church *et al.*[Bibr R53]** [54]	2018	USA	Before and after	56 Urine analysis test pre-intervention, 10 urine analysis test post-intervention	Not recorded	Urine	UTI	Restricted access (soft stop)	Pre-analytical	Rates of urine testing in catheterized patients per 1000 catheter days
**Claeys *et al.*[Bibr R54]** [55]	2021	USA	Quasi-experimental	224 573 Urine cultures (UCs) from 50 901 admissions in 24 759 unique patients	Inpatients (level of care not specified)	Urine	UTI	Automated reflex testing	Post-analytical	Rate of UCs performed per 1000 PDs and the rate of Gram-negative bloodstream infection per 1000 PDs
**Dbeibo *et al.*[Bibr R55]** [56]	2020	USA	Before and after	192 Tests ordered, 57 during baseline, 71 during implementation, 64 during sustainment	Tertiary care	Stool	*C.difficile*	Restricted access (soft stop)	Pre-analytical	Number of CDI tests performed, appropriateness of CDI testing (inappropriate if the patient had <3 stools in 24 h in the absence of signs and symptoms of colitis or toxic megacolon, appropriate if a patient had ≥3 Bristol class 6 or 7 stools in 24 h in the presence of signs and symptoms of colitis), delayed testing (CDI-compatible diarrhoea based on the algorithm if the test was ordered >24 h after they met the criteria for testing), development of toxic megacolon
**Deming[Bibr R56]** [57]	2020	USA	Before and after	213 HO CDI	Not recorded	Stool	*C.difficile*		Unclear	Number of HO-CDI cases, NHSN Standardized Infection Ratio
**Doll *et al.*[Bibr R57]** [58]	2020	USA	Interrupted time series	Not recorded	Tertiary care	Stool	*C.difficile*	Restricted access (soft stop)	Pre-analytical	HO-CDI rates per 10 000 PDs, number of inpatient tests performed per 10 000 PDs, community-associated *C. difficile* infection rates per 10 000 patient visits
**Dougherty *et al.*[Bibr R58]** [59]	2020	USA	Before and after	Not recorded	Tertiary care	Urine	UTI	Restricted access (soft stop) to test via decision support tool and standardized specimen collection and automated reflex testing	Pre-analytical, analytical and post-analytical	Urine culture and urinalysis orders per 1000 patient days, catheter-associated urinary tract infection rate per 1000 catheter days and urine culture contamination rate per 1000 PDs.
**Elkafrawy *et al.*[Bibr R59]** [60]	2019	USA	Before and after	Not recorded	Not recorded	Stool	*C.difficile*	Laboratory assessment of test appropriateness	Pre-analytical	Total CDI tests performed, patients who had initial test orders cancelled by the lab and then a second order which met the criteria for testing in the same hospital admission, completed CDI test positive/negative results
**Fabre *et al.*[Bibr R60]** [61]	2020	USA	Quasi-experimental	4315 Patients (3802 intervention group, control group 513)	Secondary care	Blood	BCs	Restricted access (soft stop)	Pre-analytical	Blood culture utilization rate (100 PDs), proportion of inappropriate BCs, proportion of positive BCs, compliance with the blood culture component of the SEP-1 measure
**Fabre *et al.*[Bibr R61]** [62]	2020	USA	Before and after	266 UCs	Secondary care	Urine	UTI	Restricted access (soft stop)	Pre-analytical	Urinary culture (UrCx) per 100 PDs, inappropriate UrCx and the number of catheter-associated UTIs
**Fabre *et al.*[Bibr R62]** [63]	2019	USA	Before and after	Not reported (N/R)	Secondary care	Urine	UTI	Restricted access (soft stop)	Pre-analytical	Mean rate of UCx orders per 100 patients, proportion of inappropriate UCs, asymptomatic bacteriuria inappropriately treated
**Farrell *et al.***[64]	2019	USA	Before and after	383 *C. difficile* test	Secondary care	Stool	*C.difficile*	Restricted access (soft stop)	Pre-analytical	Proportion of inappropriate requested tests, proportion of appropriate tests, number of cases
**Friedland *et al.*[Bibr R64]** [65]	2019	USA	Before and after	280 Patients	Secondary care	Stool	*C.difficile*	Restricted access (soft stop)	Pre-analytical	Positive *C. difficile*, ≥3 loose stools per day test, any loose stool plus abdominal cramping, clinically significant diarrhoea, testing done as part of B sepsis panel
**Gupta *et al.*[Bibr R65]** [66]	2022	India	Before and after	6044 Blood culture bottles (1028 baseline samples and 5016 end of development samples)	Tertiary care	Blood	BCs	Access to an improved diagnostic test	Pre-analytical, analytical and post-analytical	Time-points of various steps in test procedure: time to positivity, time to final report
**Halabi *et al.*** [67]	2022	USA	Interrupted time series	1056 *Clostridioides difficile* testing (estimated based on the median number of observations per month)	Tertiary care	Stool	*C.difficile*	Restricted access (soft stop)	Pre-analytical	Number of tests ordered, number of samples sent, overall number of positive tests and number HO-CDI cases
**Hayes *et al.*** [68]	2018	USA	Before and after	N/R	Tertiary care	Stool	*C.difficile*	Restricted access (soft stop)	Pre-analytical	HO-CDI incidence (CDI/10 000 PDs), number of alerts were overridden
**Howard-Anderson *et al.*[Bibr R68]** [69]	2020	USA	Before and after	N/R	Tertiary care	Urine	UTI	Restricted access (soft stop) and laboratory rejection of inappropriate samples and education of requesters and automated reflex testing	Pre-analytical and post-analytical	Rates of non-reflex and total UCs ordered per 1000 PDs, change in UTIs per 1000 PDs, diagnostic efficiency (proportion of UCs with bacterial growth)
**Jakharia *et al.*[Bibr R69]** [70]	2018	USA	Before and after	646 CDI orders	Not recorded	Stool	*C.difficile*	Restricted access (hard stop)	Pre-analytical	Number of tests requested, number of tests meeting test criteria, number of positive tests, HO-CDI rate
**Kang *et al.*[Bibr R70]** [71]	2020	USA	Before and after	N/R	Secondary care	Stool	*C.difficile*	Restricted access (soft stop)	Pre-analytical	Number of GI panels completed after 2 days of admission, repeat CD tests within 7 days of a prior result and all completed CD tests resulting after 3 days of admission per quarter, HO-CDI cases normalized to 10 000 PDs.
**Karaba *et al.*[Bibr R71]** [72]	2019	USA	Before and after	1030 CSF samples tested for West Nile virus	Secondary care	CSF	CNS infection (West Nile virus)	Restricted access (hard stop)	Pre-analytical	Average number of tests performed per month (nucleic acid amplification test (NAATs), immunoglobulin M antibody capture enzyme-linked immunosorbent test), test spending per month, number of positive tests
**Karlovich *et al.*[Bibr R72]** [73]	2022	USA	Quasi-experimental	13,806 CDI tests	Tertiary care	Stool	*C.difficile*	Restricted access (hard stop)	Pre-analytical	Rates of *C. difficile* testing performed (per 1000 PDs), rates of CDI were calculated as cases per 10 000 PDs, number of times the laxative alert fired, test order, completion and result
**Kendall *et al.*[Bibr R73]** [74]	2020	USA	Before and after	180 Tests	Tertiary care	Blood	1,3-β-d-Glucan test	Restricted access (soft stop)	Pre-analytical	1,3-β-d-Glucan tests performed per year, cost savings
**Khuvis *et al.*[Bibr R74]** [75]	2022	USA	Before and after	3004 Tests were ordered over 325 010 inpatient days	Tertiary care	Stool	*C.difficile*	Restricted access (soft stop)	Pre-analytical	Total orders per 1000 inpatient days, total NAATs per 1000 inpatient days, proportions of test results and test results per 1000 inpatient days
**Koll *et al.*[Bibr R75]** [76]	2017	USA	Before and after	N/R	Secondary care	Stool	*C.difficile*	Restricted access (soft stop)	Pre-analytical	Number of tests and percentage of positive tests (10 000 PDs)
**Koontz *et al.*[Bibr R76]** [77]	2021	USA	Before and after	64 165 Blood samples	Paediatric tertiary care	Blood	BCs	Restricted access (soft stop)	Pre-analytical	Site-specific blood culture rate per 100 PDs, number of delays in obtaining blood culture
**Kressel *et al.*[Bibr R77]** [78]	2021	USA	Before and after	1882 Orders	Tertiary care	Respiratory	Respiratory pathogens	Restricted access (soft stop)	Pre-analytical	Number of respiratory pathogens panel ordered, appropriateness respiratory pathogens panel ordered
**Kueht *et al.*[Bibr R78]** [79]	2022	USA	Before and after	370 Tests were ordered on 172 patients, and 166 orders were cancelled	Tertiary care	Stool	*C.difficile*	Restricted access (soft stop)	Pre-analytical	Number of *C. difficile* test ordered per quarter, number of patients treated, DOT per thousand PDs
**Kuhn *et al.*[Bibr R79]** [80]	2019	USA	Before and after	341 Tests	Secondary care	Stool	*C.difficile*	Restricted access (soft stop)	Pre-analytical	Bed-days of care, total number of tests ordered, number of positive tests and use of oral vancomycin was collected
**Kwon *et al.*[Bibr R80]** [81]	2019	USA	Before and after	1146 Admissions with *C. difficile* tests	Secondary care	Stool	*C.difficile*	Restricted access (hard stop)	Pre-analytical	Testing rates per 100 admissions, percentage of positive assays, number of repeat tests, mean number of tests per admission, patient discharge location, patients who received the CDI ICD-9 code, all-cause death within 30 days or colectomy due to CDI, number of patients on an antibiotic targeting *C. difficile*
**Lakshmi *et al.*[Bibr R81]** [82]	2019	USA	Before and after	2046 *C. difficile* tests	Tertiary care	Stool	*C.difficile*	Restricted access (hard stop)	Pre-analytical	*C.difficile* LabID events per 10 000 PDs, lab cancellations, HO-CDI surveillance events
**LaPorte *et al.*[Bibr R82]** [83]	2020	USA	Before and after	N/R	Secondary care	Stool	*C.difficile*	Restricted access (soft stop)	Pre-analytical	Number of HO-CDI
**Lau *et al.*[Bibr R83]** [84]	2020	USA	Random controlled trial?	187 Test orders	Tertiary care	Stool	*C.difficile*	Restricted access (soft stop)	Pre-analytical	Percentage of orders completed
**Lee *et al.*[Bibr R84]** [85]	2021	Hong Kong	Before and after	12 282 Unique UCs	Secondary care	Urine	UTI	Automated reflex testing and withholding of laboratory results in the absence of appropriate clinical details and laboratory rejection of inappropriate samples	Pre-analytical, analytical and post-analytical	Seven days of urine culture collection per patient episode, patient mortality, bacteraemia in patients with positive but unreported urine culture, CDI, emergence of MDROs and economic implications on laboratory finances
**Lennon *et al.*[Bibr R85]** [86]	2021	Australia	Before and after	192 Referrals	Tertiary care	Not stated	16S rRNA sequencing	Laboratory assessment of test appropriateness	Pre-analytical	Cost per positive 16S rRNA test, testing turnaround times
**Lin *et al.*[Bibr R86]** [87]	2019	USA	Before and after	2300 Catheter urines	Adult ICU	Urine	CA-UTI	Restricted access (soft stop)	Pre-analytical	Mean guideline adherence rate for catheterized ICU patients
**Liu *et al.*[Bibr R87]** [88]	2020	USA	Interrupted time series	Pre-intervention period = 6053 (145.1 tests/10 000 PDs). Post-intervention period = 1812 (69.8 tests/10 000 PDs).	Inpatients (level of care not specified)	Stool	*C.difficile*	Restricted access (hard stop)	Pre-analytical	*C.difficile* test orders/1000 PDs, proportion of *C. difficile* PCR test orders obtained within 48 h of laxative use, proportion of positive *C. difficile* tests
**Luu *et al.*[Bibr R88]** [89]	2021	USA	Before and after	Baseline requests = 509 per month. Post-intervention = 280	Inpatients (level of care not specified)	Urine	CA-UTI	Education	Pre-analytical	Total catheter urine culture requests per 1000 PDs
**Madden *et al.*[Bibr R89]** [90]	2019	USA	Interrupted time series	769 patients	Tertiary care	Stool	i	Restricted access (soft stop)	Pre-analytical	*C.difficile* tests/1000 PDs, LabID CDI events/10 000 bed days, duplicate negative results/10 000 PDs, duplicate positive results <14 days apart/10 000 PDs, rate of laboratory rejection of stool samples/10 000 PDs, all-cause mortality/10 000 PDs
**Madden *et al.*[Bibr R90]** [29]	2018	USA	Before and after	Pre-intervention 233 577 PDs, post-intervention 132 641 PDs	Tertiary care	Stool	*C.difficile*	Restricted access (soft stop)	Pre-analytical	*C.difficile* PCR test requests/1000 PDs, HO-CDI events/1000 PDs, duplicate negative tests/10 000 PDs, duplicate positive tests/10 000 PDs
**Marcelin *et al.*[Bibr R91]** [91]	2019	USA	Before and after	Pre-intervention: 1587 gastrointestinal pathogen panel (GIPP) tests (7.48 per 1000 PDs). Post-intervention: 1165 GIPP tests (5.24 per 1000 PDs)	Tertiary care	Stool	Gastrointestinal pathogens	Restricted access (hard stop)	Pre-analytical	GIPP orders/1000 PDs, total cost savings ($)
**Marchand-Senecal *et al.*[Bibr R92]** [92]	2021	Canada	Before and after	656 Swab specimens	Inpatients (level of care not specified)	Wound swab	Skin and soft tissue infections (SSTIs)	Laboratory rejection of poor-quality samples	Analytical	Proportion of patients with reflexive antibiotic initiation, inpatient antibiotic DOT during hospital stay and the proportion of patients with antibiotic discontinuation by day 5
**Messaca *et al.*[Bibr R93]** [93]	2022	USA	Before and after	1127 Post-implementation cases	Quaternary paediatric care	CSF	CNS infection	Restricted access (hard stop)	Pre-analytical	Time to optimal antimicrobials or time to antimicrobial cessation (if no treatable pathogen was identified)
**Mizusawa *et al.*[Bibr R94]** [94]	2019	USA	Before and after	N/R	Tertiary care	Stool	*C.difficile*	Restricted access (hard stop)	Pre-analytical	*C.difficile* orders/1000 inpatient days, percentage of providers following soft/hard stop BPA
**Monsalud *et al.*[Bibr R95]** [95]	2020	USA	Before and after	134 UCx	Not recorded	Urine	CA-UTI	Modification of laboratory reporting procedures	Post-analytical	Proportion of UCx diagnosing CA-UTI
**Moss *et al.*[Bibr R96]** [96]	2019	USA	Before and after	300 patients	Not recorded	Stool	*C.difficile*	Education of test requestors and automated reflex testing	Pre-analytical and post-analytical	Proportion of patients on appropriate CDI treatment
**Munigala *et al.*[Bibr R97]** [97]	2019	USA	Before and after	15 954 Patients (7.174 in post-intervention period)	Tertiary care	Urine	UTI	Restricted access (soft stop) and automated reflex testing and education of test requestors	Pre-analytical and post-analytical	Urine culture rate/1000 bed days
**Nanda *et al.*[Bibr R98]** [98]	2018	USA	Before and after	567 marker-Procalcitonin, PCT orders	Tertiary care	Blood	Procalcitonin	Restricted access (soft stop)	Pre-analytical	Proportion of appropriate PCT orders in pre-IP and post-IP
**Newman *et al.*[Bibr R99]** [99]	2018	USA	Before and after	678 *C. difficile* orders	Not recorded	Stool	*C.difficile*	Restricted access (hard stop)	Pre-analytical	Community-onset-CDI (CO-CDI) detection rate, percentage of appropriate *C. difficile* PCR requests
**Nix *et al.*[Bibr R100]** [100]	2021	USA	Before and after	N/R	Stem cell transplant and haematology	Stool	*C.difficile*	Restricted access (soft stop)	Pre-analytical	*C.difficile* testing volume (tests per 100 PDs), NHSN-defined *C. difficile* identifications, oral vancomycin DOT per 100 PDs, number of distinct patients with an order for oral vancomycin, number of vancomycin-resistant Enterococcus (VRE) conversions (per 100-PDs), HO-VRE bacteremias
**Page *et al.*[Bibr R101]** [101]	2020	USA	Before and after	25 UCs	Neurology ICU	Urine	CA-UTI	Agile implementation model	Pre-analytical	CA-UTI incidence rates, number of UCs or urinalyses with reflex to culture and total antibiotic use (as a surrogate to empiric tx)
**Penney *et al.*[Bibr R102]** [102]	2022	USA	Time-series analysis	787 Cultures (from power calc)	Tertiary care	Urine	UTI	Automated reflex testing	Post-analytical	The change in culture rates of tests ordered as urinalysis with reflex to culture (UARC) per 1000 PDs before the intervention and after the intervention, the change in culture positivity, which is the proportion of cultures reflexed from UARC with bacterial growth from cultures performed, UARC performance, which is the proportion of cultures reflexed from UARC from UARC ordered, secondary outcomes included [1] antimicrobials prescribed for suspected UTI per 1000 PDs and [2] CA-UTIs per 1000 urinary-catheter days
**Penney *et al.*[Bibr R103]** [103]	2022	USA	Time-series analysis	1494 Unique isolated urine culture orders	Tertiary care	Urine	UTI	Restricted access (soft stop)	Pre-analytical	The change in isolated urine-culture rates per 1000 PDs between the pre-intervention period and the post-intervention period. Secondary outcomes: measures of testing utilization including the change in culture positivity (proportion of isolated UCs with bacterial growth) and isolated urine culture orders as a proportion of all urine testing (which includes both UARC and isolated urine culture)
**Poelman *et al.*[Bibr R104]** [104]	2020	The Netherlands	Before and after	492	Emergency department	Respiratory	Respiratory infections	Access to an improved diagnostic test	Analytical	Reduced TAT, reduced ‘Euro-hour’ (cost of test/TAT)
**Prodanuk *et al.*** [105]	2020	Canada	Before and after	2183	Paediatric emergency department	Urine	UTI	Restricted access (soft stop) to test via decision support tool. Call-back service with the result	Pre-analytical and post-analytical	20% Reduction is misdiagnosis. 30% Reduction in antibiotic duration. 78.9% Algorithm adherence. Callback system allowed increase of antibiotic discontinuation from 0% to 76.8%, with 1678 antibiotic days saved. 8/106 Patients with positive culture and missed UTI diagnosis re-presented within 72 h, and two required admission and IV antibiotics
**Qamar *et al.*[Bibr R106]** [106]	2021	USA	Before and after	238	Inpatients (level of care not specified)	Stool	*C.difficile*	Restricted access (soft stop)	Pre-analytical	No reduction in inappropriate tests as users over-rode the order set
**Qutaishat *et al.*[Bibr R107]** [107]	2018	USA	Before and after	Not stated	Inpatients (level of care not specified)	Stool	*C.difficile*	Laboratory assessment of test appropriateness	Pre-analytical	Reduction in HO-CDI rate, standardized infection ratios, vancomycin use and contact precaution days
**Rico *et al.*[Bibr R108]** [108]	2021	USA	Before and after	120	Inpatients (level of care not specified)	Urine	UTI	Education of test requestors and automated reflex testing	Pre-analytical and post-analytical	Reduction in number of patients treated for ASB > no reduction in LOS. Reduction in length of treatment. Reduction in number of urinalyses and cultures performed
**Rock *et al.*[Bibr R109]** [109]	2022	USA	Before and after	Not stated	Inpatients (level of care not specified)	Stool	*C.difficile*	Restricted access (hard stop)	Pre-analytical	Reduction in *C.difficile* testing rates. Reduction in CDI rates. Reduction in Abx used to treated CDI
**Schinkel *et al.*[Bibr R21]** [21]	2022	The Netherlands and USA	Before and after	8027 In training, 36 096 in validation	Emergency department	Blood	BCs	Machine learning	Pre-analytical	The model could already prevent over 30% of BCs, while missing a true-positive culture in 1% of cases
**Schultz *et al.*[Bibr R110]** [110]	2018	USA	Before and after	Not stated – study run across whole institution over 13 months	Inpatients (level of care not specified)	Stool	*C.difficile*	Restricted access (soft stop)	Pre-analytical	Testing only liquid stool: 1.8% increased compliance (*P* = 0.63). No test with 7 days of previous negative: 7.8% increased compliance (*P* = 0.002). No test within 14 days previous positive: 10.5% increased compliance (*P* ≤ 0.001). No test within 48 h of laxatives: 7.9% increased compliance (*P* = 0.02)
**Schwarz *et al.*[Bibr R111]** [111]	2020	USA	Before and after	271	Not recorded	Blood	Candidaemia	Access to an improved diagnostic test	Analytical	Antifungal administration within the time of assay collection was 54% in the negative group vs 74% in the positive group (*P* = 0.030). Mean duration of antifungal use was significantly lower in the negative group than in the positive group (5.98 vs 17.55 days, *P* = 0.04)
**Scott *et al.*[Bibr R112]** [112]	2022	USA	Time series	Not stated	Not recorded	Urine	UTI	Restricted access (soft stop)	Pre-analytical	23.5% Reduction in the amount of urine culture tests completed (*P* < 0.001). A 15.9% CA-UTI reduction and a 23.3% CA-UTI rate reduction
**Shallal *et al.*[Bibr R113]** [113]	2022	USA	Before and after	Not stated. Pre-intervention Jan 2018–Dec 2019. Post-intervention Apr 2020–Sep 2021	Inpatients (level of care not specified)	Stool	*C.difficile*	Restricted access (hard stop)	Pre-analytical	CDI rates per 1000 PDs reduced by 54% (3.21–1.48). Test order rates were reduced by 26.5% (119.4–87.7) The standardized infection ratio decreased by 33% (0.542–0.361)
**Shields *et al.*[Bibr R114]** [114]	2018	USA	Before and after	88	ICU	Blood	Candidaemia	Access to an improved diagnostic test	Analytical	47% Reduction in antifungal use (no control stated)
**Sick-Samuels *et al.*[Bibr R115]** [115]	2019	USA	Time series	Not stated. One year pre-inervention periods (to April 2018), and 1 year post-intervention	Paediatric ICU	Endotracheal aspirate	Respiratory pathogens	Restricted access (hard stop)	Pre-analytical	In the pre-intervention period, there was an average of 46 ETA cultures/month, a total of 557 cultures over 5092 ventilator days. After introduction of the algorithm, there were 19 cultures/month, a total of 231 cultures over 3554 ventilator days (incident rate 10.9 vs 6.5 per 100 ventilator days – a 43% decrease). The intervention led to an estimated $6000 in monthly charge savings
**Solanky *et al.*[Bibr R32]** [33]	2021	USA	Before and after	Not stated	Not recorded	Stool	*C.difficile*	Restricted access (soft stop)	Pre-analytical	Reduction in number of NAATs, HO-CDI cases, CDI Rx costs
**Sperling *et al.*[Bibr R116]** [116]	2019	USA	Before and after	Not stated	Not recorded	Stool	*C.difficile*	Restricted access (hard stop)	Pre-analytical	Testing rate was reduced by 42% (198–115 tests per 10 000 PDs). HO-CDI LabID event rates decreased by 59%(12.3–5.0 cases per 10 000 PDs). The HO-CDI LabID standardized infection ratio decreased from 1.017 in 2016 to 0.699. Days of oral vancomycin in hospitalized patients decreased by 27% (7.3–5.3 per 1000 PDs)
**Sterling *et al.*[Bibr R117]** [117]	2019	USA	Before and after	Not stated	Not recorded	Stool	*C.difficile*	Restricted access (hard stop)	Pre-analytical	HO-CDI rates fell from 0.75 to 0.48 cases per 1000 PDs, with an estimated costs savings of $259 555 per quarter and $1.04 million per year. *C. difficile* PCR guideline compliance increased from 39% to 53%; orders decreased by 50% post-intervention
**Sullivan *et al.*[Bibr R118]** [118]	2020	USA	Before and after	Pre-intervention: 2150 samples. Post-intervention: 22 028 samples	Tertiary care	Stool	*C.difficile*	Automated reflex testing	Post-analytical	A reduction in the percentage and number of CDI diagnoses among indeterminate cases: 182 of 281 (65%) in the pre-intervention group and 94 of 219 (43%) in the post-intervention group (*P* < 0.01). Significantly fewer patients with indeterminate EIA results had PCR testing performed in the post-intervention vs pre-intervention group (66% vs 99.6%; *P* < 0.001). No significant differences in the number of patients treated for CDI between the two groups. For the 276 patients with a positive PCR, those in the post-intervention period were more likely to be treated: 87% in the pre-intervention period vs 95% in the post-intervention period (*P* = 0.04). They also had a slightly longer antibiotic duration, with a median of 14 days (range: 0–49) in the pre-intervention period vs 14 days (range: 0–66) in the post-intervention period (*P* = 0.04). Both groups had similar frequencies of adverse outcomes. The potential cost avoidance with each reflexive PCR not performed per patient was calculated to be $4498. This cost included the PCR test ($296), a 14-day course of oral vancomycin 125 mg every 6 h ($119), isolation supply cost for 5 days ($250), and one additional day of hospital admission ($3833) since patients without a PCR performed had a median of one fewer day of hospitalization
**Tai *et al.*[Bibr R119]** [119]	2021	USA	Before and after	2641 Samples (1560 pre, 1073 post)	Inpatients (level of care not specified)	Stool	Gastrointestinal pathogens	Restricted access (hard stop)	Pre-analytical	Reduction in the number of PCRs performed. No significant decrease in proportion of positive test results in the post-intervention phase
**Tchou *et al.*[Bibr R120]** [120]	2020	USA	Before and after	Not stated. 700 Bed centre, with 2500 annual admission to the 35 bed pediatric intensive care unit (PICU)	Quaternary paediatric care	Blood	Blood gases	Education	Pre-analytical	After initiating provider training, mean testing rates decreased from 0.94 to 0.60 point-of-care (POC) tests per PICU PD. With further training, we observed a brief reduction to 0.23 POC blood gas tests per PICU PD. After a formalized practice guideline was created and implemented and training was incorporated into existing resident orientation structures, testing rates stabilized at 0.41 tests per PD. Variation in testing rates decreased after implementing systematic training. Based on a sustained post-intervention testing rate of 0.41 POC blood gas tests per PICU PD, we calculated the total estimated direct supply cost savings to be $19 068 per year, with estimated reduced annual potential patient charges of ≈$1.2 million
**Tirupathi *et al.*[Bibr R121]** [121]	2020	USA	Before and after	Not stated	Inpatients (level of care not specified)	Stool	*C.difficile*	Restricted access (hard stop) and automated reflex testing	Pre-analytical and post-analytical	34% Reduction in PCR tests (940–626). 28% Reduction of HO-CDI (60–43), further reduction to 28 the following year. 54% Reduction overall. $8300 Lab cost savings
**Touzard-Romo *et al.*[Bibr R122]** [122]	2021	USA	Before and after	72 Samples	Inpatients (level of care not specified)	Stool	*C.difficile*	Restricted access (hard stop)	Pre-analytical	A total of 72 samples required CD testing authorization; 65 (90%) were approved. Baseline demographics, in-hospital death and LOS were similar in both groups, but approved patients were four times as likely to have ≥3 loose stools in 24 h compared to not approved. The number of CD tests was 13 at baseline with a decrease of 6 tests in the first month of intervention (95% CI: −10.0, −1.35), followed by an insignificant decline in the monthly trend (−0.14; 95% CI: −0.49, 0.20). There were 22 HO-CDI pre-intervention and 10 post-intervention. Pre-intervention, incidence of HO-CDI was 0.51 cases × 1000 PD and increased every month by 0.11 (95% CI: 0.07, 0.16). In July 2019, there was a significant decline of 1.16 case × 1000 PD (95% CI: −1.99, −0.33), followed by monthly decline (−0.16; 95% CI: −0.23, −0.09). Our calculated standard infection ratios after the intervention decreased to 0.77 from 1.03
**Tran *et al.*[Bibr R123]** [123]	2020	USA	Before and after	228 Test requests	Tertiary care	Stool	*C.difficile*	Restricted access (hard stop)	Pre-analytical	An average of 2.5 requests per day was received over the 3-month intervention period. The weekly rate of EIA and GIPAN orders per 1000 PDs decreased significantly from 6.05 ± 0.94 to 4.87 ± 0.78 (IRR, 0.72; 95% CI, 0.56–0.93; *P* = 0.010) and from 1.72 ± 0.37 to 0.89 ± 0.29 (IRR, 0.53; 95% CI, 0.37–0.77; *P* = 0.001), respectively. 57 HO-CDI cases (including 3 cases in paediatric patients) occurred during the pre-intervention period, and 39 (no paediatric cases) occurred during the intervention period, representing a 32% absolute reduction. Overall, the weekly rate per 10 000 PDs was 5.57 ± 1.39 in the pre-intervention group and 3.92 ± 2.46 in the intervention group (IRR, 0.74; 95% CI, 0.49–1.11; *P* = 0.148)
**Turner *et al.*[Bibr R124]** [124]	2019	USA	Before and after	Not stated	Secondary care	Stool	*C.difficile*	Restricted access (soft stop)	Pre-analytical	Overall reduction in CDI. Reduction in number of positive tests
**van den Bergh *et al.*[Bibr R125]** [125]	2020	South Africa	Before and after	2464 Patients in 39 hospitals were included in the final analysis	Inpatients (level of care not specified)	Blood and Sputum	Respiratory pathogens	Restricted access (soft stop)	Pre-analytical	Diagnostic stewardship compliance improved overall from 49·1% to 54·6% (CI 3·3–7·7, *P* < 0·0001). Culture compliance improved from 185/1247 (14.8%) to 269/1217 (22.1%). There was no difference in mortality between the two phases [4·4%(55/1247) vs 3·9% (47/1217); *P* = 054], median LOS or IR LOS 6.0 vs 6.0 days (*P* = 0·20) and 5.0 vs 5.0 days (*P* = 0·40)
**Waagsbo *et al.*[Bibr R126]** [126]	2022	Norway	Before and after	1280	Inpatients (level of care not specified)	Respiratory and blood	Respiratory pathogens		Unclear	Various diagnostic yield data detailed in the paper
**Wadskier Montagne *et al.*** [127]	2019	USA	Before and after		Not recorded	Stool	*C.difficile*	Withholding results until patient review by expert	Post-analytical	Of 274 patients with a GIP *C. difficile* (+) result, 71 (26%) had no SOC sent. Of positive SOCs, 45 patients (37%) were positive by toxin A/B enzyme immunoassay (EIA) and 77 (63%) by PCR only. There were 2153 total SOC tests sent; of these, 332 (15%) were positive; 130 (39%) by toxin and 202 (61%) by PCR only. Mortality and 30-day re-admission were not significantly different between groups. CDI rates within 3 months were not significantly different between GIP (+) only and control group (*P* = 0.11). In contrast, those with SOC (+) tests had more true CDI within 3 months, compared with controls (*P* < 0.001)
**Walker *et al.*[Bibr R128]** [128]	2022	Germany	Before and after	295 (Pre-intervention 137, post-intervention 158)	Adult ICU	Blood	BCs	Education	Pre-analytical	From pre- to post-interventional, the number of ≥2 culture sets per episode increased from 63.9% (257/402) to 81.3% (230/283), and venipunctures increased from 42.5% (171/402) to 77.4% (219/283). The positivity rate decreased from 15.1% (108/714) to 12.8% (83/650), as did the contamination rate (3.8%–3.6%). The majority of the aerobic bottles were filled within the target range (255/471, 54.1%), but in 96.6%, the anaerobic bottles were overfilled (451/467)
**Walter *et al.*[Bibr R129]** [129]	2022	USA	Before and after	Not stated	Inpatients (level of care not specified)	Stool	*C.difficile*	Restricted access (hard stop)	Pre-analytical	Reduction of HO-CDI from 12 to 4.72/10 000 PDs after year 1 to 2.8/10 000 PDs in years 2–4. 54% Incresae in testing on days 1–3 of admission. 50% Decrease in testing on days 4+
**Wang *et al.*[Bibr R130]** [130]	2021	USA	Before and after	Not stated	Inpatients (level of care not specified)	Stool	*C.difficile*	Restricted access (hard stop)	Pre-analytical	Reduction of HO-CDI from 5.02 to 1.64/10 000 PDs. Reduction in tests from 90.38 to 76.64/10 000 PDs. Reduction in positive tests from 15.46% to 5.66%. Assessment of test appropriateness increased from 48% to 58%. Reduction of VANC use from 439.73 to 394.38 DOT/1000 days. No reduction in fidaxomicin use. Estimated annual saving of $794 150
**Watson *et al.*[Bibr R131]** [131]	2020	USA	Before and after	Not stated	Inpatients (level of care not specified)	Urine	UTI	Restricted access (hard stop) and automated reflex testing	Pre-analytical and post-analytical	Following implementation of the new order set, the number of UCs performed among the five sites decreased from 1175.8 tests per 10 000 PDs before the intervention to 701.4 after the intervention (40.4% reduction; *P* < 0.01). Antibiotic DOT for patients with a urinary tract infection indication decreased from 102.5 to 86.9 per 1000 PDs (15.2% reduction; *P* < 0.01). The CA-UTI standardized infection ratio was 1.0 before the intervention and 0.8 after the intervention (*P* = 0.23). The estimated yearly savings following the intervention was US$535 181.
**Woods-Hill *et al.*[Bibr R132]** [132]	2022	USA	Before and after	Not stated	Paediatric ICU	Blood	BCs	Restricted access (soft stop)	Pre-analytical	Across the 14 PICUs, the blood culture rate was 149.4 per 1000 PDs/month pre-implementation and 100.5 per 1000 PDs/month post-implementation, for a 33% relative reduction (95% CI, 26%–39%). Comparing the periods before and after implementation, the rate of broad-spectrum antibiotic use decreased from 506 to 440 days per 1000 PDs/month, respectively, a 13% relative reduction (95% CI, 7%–19%). The broad-spectrum antibiotic initiation rate decreased from 58.1 to 53.6 initiations/1000 PDs/month, an 8% relative reduction (95% CI, 4%–11%). Rates of CLABSI decreased from 1.8 to 1.1 per 1000 central venous line days/month, a 36% relative reduction (95% CI, 20%–49%). Mortality, LOS, re-admission, sepsis and severe sepsis/septic shock were similar before and after implementation
**Woods-Hill *et al.*** [133]	2018	USA	Before and after	Not stated	Paediatric ICU	Blood	BCs	Restricted access (soft stop)	Pre-analytical	Blood culture rates decreased from 13.3, 13.5 and 11.5 cultures per 100 PDs pre-implementation to 6.4, 9.1 and 8.3 cultures per 100 PDs post-implementation for unit A, B and C, respectively; a decrease of 32% (95% CI, 25%–43%; *P* < 0.001) for the three units combined. Post-implementation, the proportion of total BCs drawn from central venous catheters decreased by 51% for the three units combined (95% CI, 29%–66%; *P* < 0.001). Notable difference between units included the identity and involvement of the project champion, adaptions of the clinical tools and staff monitoring and communication of project progress. Qualitative data also revealed a core set of barriers and facilitators to behaviour change around paediatric intensive care unit blood culture practices
**Woods-Hill *et al.*[Bibr R134]** [134]	2018	USA	Before and after	Not stated	Paediatric ICU	Blood	BCs	Not stated	Unclear	1103 Cultures in 8301 PDs (IR 13.3 per 100 PDs) vs 398 cultures in 5625 PDs (IR 7.1 per 100 PDs), corresponded to a 47% reduction in culture rate/100 PDs (IRR 0.53, 95% CI 0.48–0.60) in unit A; 2143 cultures in 15 825 PDs (IR 13.5 per 100 PDs) vs 745 cultures in 6458 PDs (IR 11.5 per 100 PDs) corresponded to a 15% reduction in culture rate/100 PDs in unit B (IRR 0.85, 95% CI 0.78–0.93); 1611 cultures in 14 039 PDs (IR 11.5 per 100 PDs) vs 687 cultures in 7814 PDs (IR 8.8 per 100 PDs) corresponded to a 23% reduction in culture rate/100 PDs in unit C (IRR 0.77, 95% CI 0.70–0.84). There was an 8% sustained monthly decrease in culture rate per 100 PDs/month in both units A and B (IRR 0.92, 95% CI 0.87–0.98, *P* = 0.009 for unit A; IRR 0.92, 95% CI 0.89–0.95; *P* < 0.001 for unit B). In unit C, an immediate drop in blood culture rate of 30% (IRR 0.70, 95% CI 0.48–1.03, *P* = 0.068) was followed by a sustained 1% decrease in culture rates per 100 PDs/month (IRR 0.99, 95% CI 0.97–1.03, *P* = 0.81)
**Wulff *et al.*[Bibr R135]** [135]	2022	USA	Before and after	14 462	Quaternary care	Respiratory	SARS CoV-2	Restricted access (hard stop)	Pre-analytical	This study included 14 462 severe acute respiratory syndrome coronavirus two reverse transcriptase PCR tests ordered during the study period. After the intervention, there was a 27.3% decrease in non-conforming rapid tests. Rapid test reporting time from laboratory receipt decreased by 1.47 h. The number of days of rapid test inventory on hand increased by 39 days
**Yen *et al.*[Bibr R31]** [32]	2018	USA	Before and after	Not stated	Tertiary care	Stool	*C.difficile*	Education	Pre-analytical	Reduction in monthly average *C. diffcile* tests from 148 to 85 (43%), with a 36% reduction in positivity. Reduction in false-positive HO-CDI from 8 to 3 per month (63%), projected monthly financial savings: $2017 in lab costs, $5000 Rx costs
**Zaver *et al.* [Bibr R136][136]**	2021	USA	Before and after	Not stated	Not recorded	Stool	*C.difficile*	Restricted access (soft stop)	Pre-analytical	83.6% Reduction in inappropriate orders tested and a 41.7% reduction in health care facility on set CDI incidence

DOT, Days of therapy; HO-CDI, hospital-onset *Clostridium difficile* infection; LOS, length of stay; PDs, patient days.

**Fig. 2.**
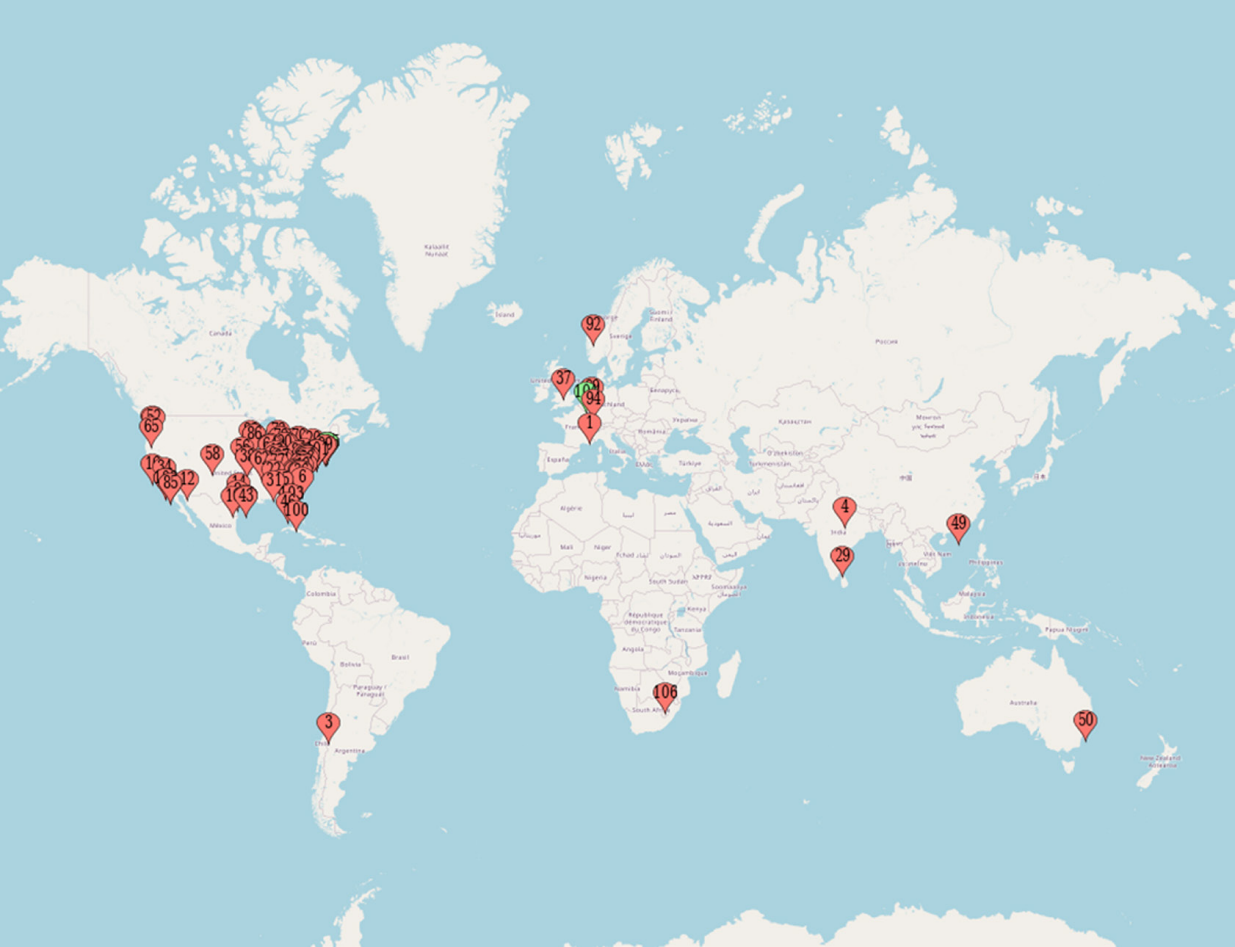
Geographical location of included studies.

As depicted in Fig. 2, the bulk of studies that characterize their interventions as diagnostic stewardship approaches were primarily conducted in the USA [*n* = 92, 87.6 % (number of studies = *n*, percentage of total number of studies)]. Following this, there were smaller numbers of such studies in Canada (*n* = 2, 1.9 %), India (*n* = 2, 1.9 %), Australia (*n* = 1, 1 %), Chile (*n* = 1, 1 %), Germany (*n* = 1, 1 %), Hong Kong (*n* = 1, 1 %), the Netherlands (*n* = 1, 1 %), the Netherlands and the USA (*n* = 1, 1 %), Norway (*n* = 1, 1 %), South Africa (*n* = 1, 1 %) and Switzerland (*n* = 1, 1%).

**Fig. 3.**
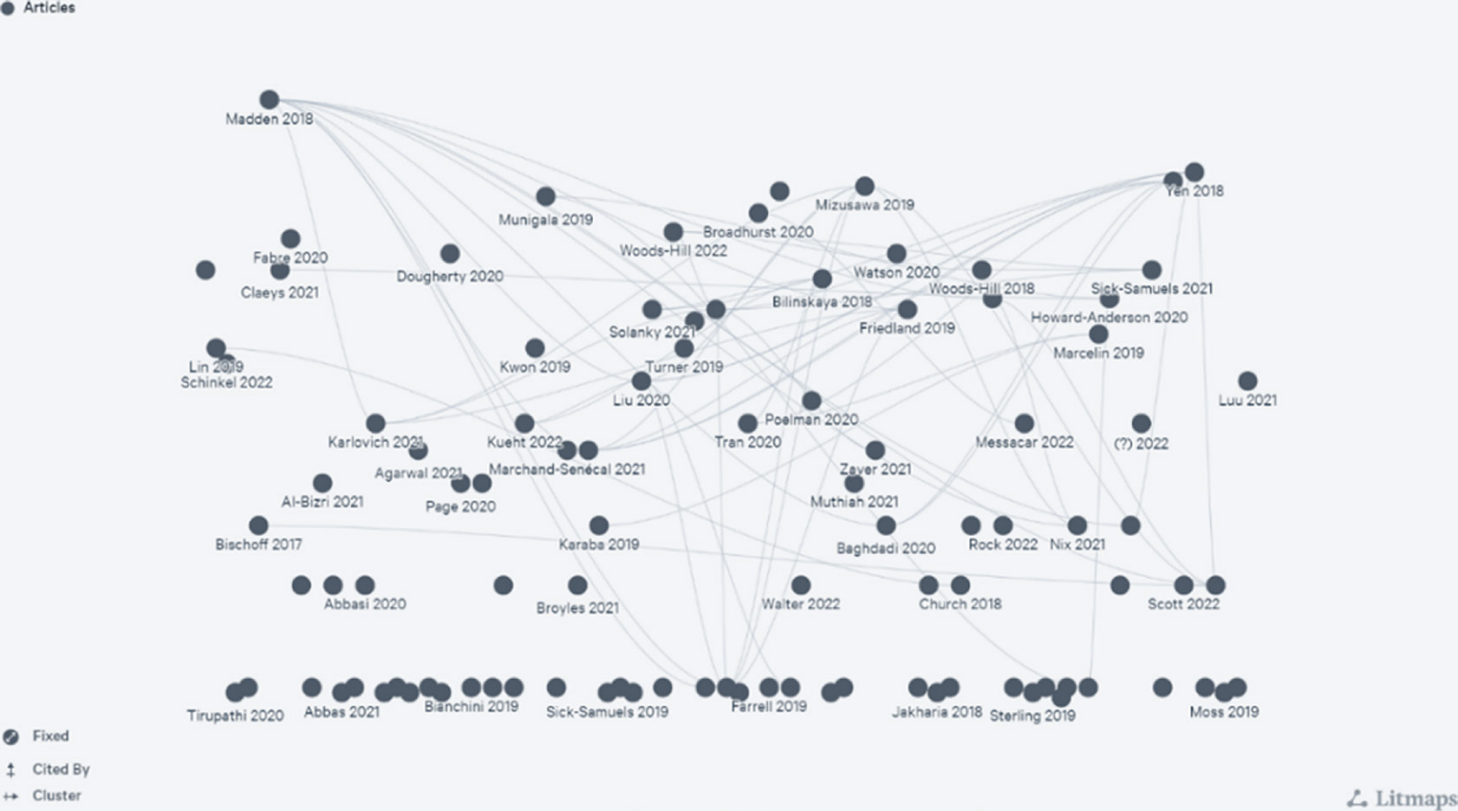
Litmaps citation map of all included studies.

The Litmaps citation map demonstrates the current body of evidence which describes their intervention as diagnostic stewardship and their citation relationship (see Fig. 3 for Litmaps citation map of all included studies). However, there are some clusters of papers which seem to be detached from the main body of evidence and do not seem to be citing the current evidence within this area. The most internally cited study was by Madden *et al*. [29], which was cited 10 times by other studies within this scoping review. A total of 38 (36.2 %) papers remained uncited by any other paper within the confines of this scoping review.

Most studies that defined their intervention as adopting a diagnostic stewardship approach employed a before and after research design (*n* = 91, 86.6 %). Regrettably, it posed a challenge to consistently determine whether the studies followed a retrospective or prospective approach; therefore, it was decided for this not to be coded. The second most prevalent study design involved an interruptive time series, in which data were gathered both before, during and after the intervention (*n* = 9, 8.6 %). A limited number of studies incorporated a control group, with only four non-randomized controlled trials and one randomized controlled trial.

Studies varied in size ranging from small studies which used 77 samples to larger studies which used 224 573 samples. Diagnostic stewardship interventions were primarily implemented in entire clinical hospitals, with 36 studies (34.3 %) conducted at tertiary care facilities and 14 (13.3 %) at secondary care facilities. Fewer studies focused on specific clinical settings, including paediatric ICU (*n* = 4, 3.8 %), adult ICU (*n* = 3, 2.9 %), emergency department (*n* = 3, 2.9%), quaternary paediatric care (*n* = 2, 1.9%), quaternary care (*n* = 1, 1 %), paediatric tertiary care (*n* = 1, 1 %), paediatric emergency department (*n* = 1, 1 %), neurology ICU (*n* = 1, 1 %), army medical facility (*n* = 1, 1 %) and stem cell transplant and haematology (*n* = 1, 1 %). The remaining studies either only indicated that the intervention was undertaken in an inpatient setting, but no specific description of care was given (*n* = 21), or the type of setting was not described (*n* = 16). Among the included studies, 70 were conducted in a single centre, while 25 studies occurred across multiple centres. In 10 studies, the number of clinical settings where the intervention took place was not specified.

Within the included studies, the most common sample types which the diagnostic stewardship intervention focused upon were stool (diagnosis of *Clostridioides difficile* infection *n* = 52, 49.5 %, infective gastroenteritis *n* = 1, 1 %), urine (urinary tract infection *n* = 20, 19%), blood (*n* = 18, 14.1 %; BCs *n* = 10, 9.5 %; procalcitonin *n* = 2, 1.9 %; candidaemia *n* = 2, 1.9 %; hepatitis C virus *n* = 1, 1 %; thyroid function tests *n* = 1, 1 %; beta-d-glucan *n* = 1, 1%; blood gases *n* = 1, 1 %) and cerebrospinal fluid samples (meningitis/encephalitis *n* = 4, diagnosis of West Nile virus *n* = 1, 1 %). The remaining studies used respiratory (respiratory infections *n* = 2, 1.9 %; SARS CoV-2 *n* = 1, 1%) blood and sputum (community-acquired pneumonia *n* = 2), endotracheal aspirate (ventilator-associated infection *n* = 1, 1 %), wound swab (skin and soft tissue infections *n* = 1, 1 %) and multiple samples (multiple infections *n* = 1, 1 %), and one study did not specify what type of sample was used.

The outcomes used to assess interventions defined as diagnostic stewardship varied but could be categorized under four main categories. Of these four categories, the most common outcome reported was test usage (*n* = 74, 70.5 %); this was typically measured either as number of tests over the data collection period or reported as an incidence rate over a given period. The second most common reported outcome was the number of positive tests (*n* = 63, 60 %); this was mainly reported as a ratio or a percentage. The joint third most recorded outcomes were patient outcomes (*n* = 32, 30.5 %); these were typically linked to the diagnostic test (e.g. in-hospital mortality, length of stay and days of therapy). Similarly, protocol/process-related outcomes were reported in 32 studies (30.5 %); these outcomes were regarding compliance to test protocol/process in both ordering the correct test and carry out the test within set criteria such as within a given period. The fourth most common outcome reported was regarding cost assessment (*n* = 15, 14.3 %), typically reported as cost saving over a given period. The least common outcome reported was sensitivity and specificity of the nearly adaptive tests protocol (*n* = 4, 3.8 %).

**Fig. 4.**
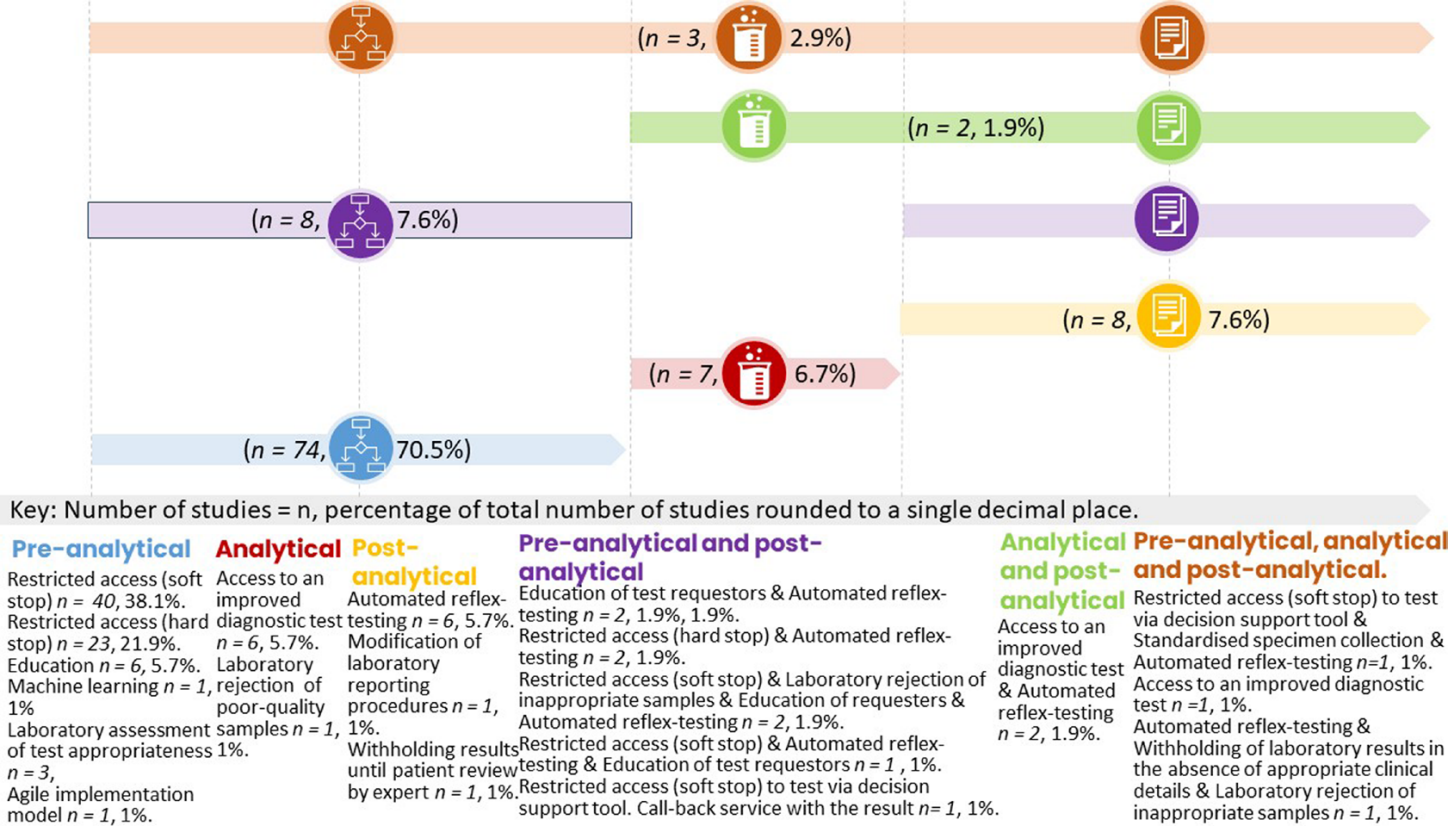
Visual representation of stage of delivery of diagnostic stewardship interventions.

As depicted in Fig. 4, the pre-analytical phase is the predominant stage for intervention instances (*n* = 74, 70.5 %). These interventions commonly follow a restrictive strategy, manifested either as a decisive halt, where clinicians are prohibited from further test requests (*n* = 40, 38.1 %), or in a more nuanced form known as a soft stop. In the latter case, a decision support tool is employed to guide and facilitate the test selection process (*n* = 23, 21.9 %). Altogether, when aggregated, these findings revealed that 60 % of the identified studies described the intervention as diagnostic stewardship, primarily adopting a restrictive approach.

**Fig. 5.**
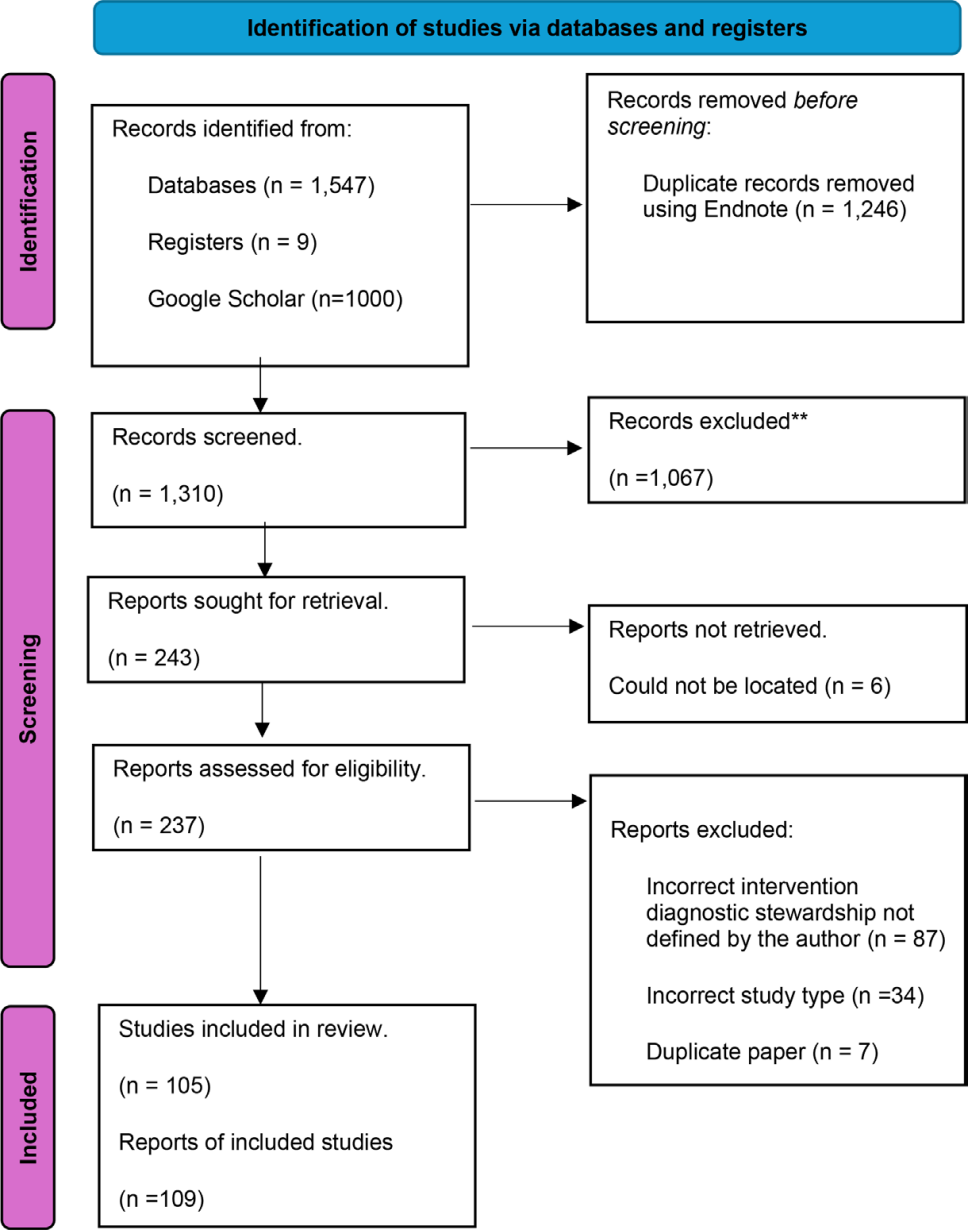
PRISMA diagram [137].

Education emerges as the third most prevalent intervention type within the pre-analytical stage (*n* = 6, 5.7 %). While a considerable portion of soft stop interventions usually involved an educational element, it was determined that education was not the primary approach employed in these instances. The remaining four studies used laboratory assessment of test appropriateness (*n* = 3, 2.9 %), machine learning (*n* = 1, 1 %) and agile implementation model (*n* = 1, 1 %)-based interventions. There were three studies which were unclear regarding the exact stage of intervention (*n* = 3, 2.9 %).

The analytical phase saw the fewest number of studies (*n* = 7, 6.7 %), with the primary strategy being the adoption of an enhanced alternative test method (*n* = 6, 5.7 %). The sole remaining study utilized an intervention around the rejection of poor-quality samples.

A minimal number of studies implemented interventions during the post-analytical stage of testing (*n* = 8, 7.6 %). The predominant approach across most studies involved automatic reflective testing interventions (*n* = 6, 5.7 %). The last two studies employed interventions involving the modification of laboratory reporting procedures and the withholding of results until patient review by expert.

A limited number of studies implemented interventions across multiple stages (*n* = 13, 12.4 %). The studies which used multiple interventions across various phases used a wide range of intervention types. In three studies, pinpointing the precise stage of intervention delivery proved challenging due to the limited level of detail provided in the intervention descriptions. A full list of classifications of each intervention stage and intervention description can be seen within Fig. 4 and Table 1.

## Discussion

In the current published literature, the term diagnostic stewardship is utilized with considerable frequency appearing in 105 papers. Despite this high prevalence of its use, the term has only been used to describe interventions taking place within studies for the last 7 years. However, it is notable that there has been a consistent increase in its use throughout this period, with this term being used in over 20 papers per year for the last 4 years. Subsequently highlighting the vogue of this term within the scholarly literature. The published literature appears to be building upon its own foundation, evidenced by the prevalent practice of mutual citation within the majority of papers. On visual inspection, there appears to be multiple citation loops suggesting a reciprocal process where studies cite and draw upon each other, contributing to a collective and evolving understanding of the subject matter. However, there still seems to be pockets of research which are not referring to previous diagnostic stewardship studies.

Despite some variation in the evidence base, it is apparent that there are common characteristics that may determine people’s understanding of the concept of diagnostic stewardship and its application in practice. Diagnostic stewardship appears to have been adopted mainly in the USA, where it is utilized as a rather unsubtle instrument to curtail the rigours of clinical testing. This is evident through the stages of intervention and the subsequent reported outcomes. More than 60 % of all studies that identified their intervention as employing diagnostic stewardship utilized a restrictive approach (hard or soft stop). Furthermore, the emphasis on process outcomes, specifically the sheer quantity of tests conducted and the tally of negative results, dominates the discourse. Other aspects, such as improved patient outcomes and reduced patient harms of inappropriate testing, were only cited in a third of studies. The environmental impact of unnecessary testing was absent. Regrettably, the absence of supplementary data reporting the sensitivity and precision of the testing process, coupled with a dearth of comprehensive insights into patient outcomes, leaves the concept of diagnostic stewardship largely unrealized. In deviating from its intended use as defined by the WHO, diagnostic stewardship has become a basic tool employed not to improve but to limit the amount of testing undertaken [23]. Therefore, future research aiming to incorporate the principles of diagnostic stewardship should take a more holistic approach by integrating the concepts of timeliness, process efficiency and efficient patient care [23].

Regarding the area of application of diagnostic stewardship interventions, this scoping review reveals that the term diagnostic stewardship is used predominantly in the field of infectious diseases rather than other pathology disciplines. This may be due to the overlapping concept of antimicrobial stewardship, and its common aim is to utilize resources appropriately [30]. It is somewhat surprising that none of the papers returned in the searches are related to diagnostic stewardship of SARS-CoV-2 testing during the COVID-19 pandemic, especially considering the vast numbers of tests performed globally. This may be related to the individual governmental drives to increase testing and early calls from WHO to ‘test test test test’ [31].

It is clear that the foundational principles regarding the timeliness and appropriateness of testing are not routinely being employed, highlighting the focus on restriction rather than enhancement. This phenomenon may, in part, be attributed to the influence of specific local or national policies on the trajectory of diagnostic stewardship interventions. For example, several studies looking to improve the diagnosis of *Clostridium difficile* infection (CDI) in the USA noted the financial penalties that an institution would be liable for [32]. In other studies, the focus was on reducing the incorrect diagnosis of hospital-onset CDI, for which care would not be reimbursable [33] rather than focusing on the enhancement of the test protocol itself. With a focus that is mainly on the field of infectious diseases, the current approaches of diagnostic stewardship exhibit alignment with ‘demand optimization’, which is a term currently used within the UK initiative ‘Getting It Right First Time’ [34]. Similarly, the approach described as ‘Choosing Wisely’ in the USA seems to be similarly aligned with the current interpretation of diagnostic stewardship, where healthcare workers choose only the tests that are supported by evidence, are not duplicative, are free from harm and are truly necessary [35]. Therefore, despite the WHO’s advocacy for an ideology that champions a more comprehensive approach to quality improvement, it may be more fitting to consider alternative regional narratives when categorizing these types of quality improvement interventions.

From a methodological perspective, studies describing interventions as diagnostic stewardship were often undermined by specific limitations in their study design, which should be addressed in future research. Given that most studies adopted a before and after research design, it was evident that very few used a control group as a comparator and even fewer used random allocation. These limitations increased any uncertainties in the findings. It is important for researchers to direct their efforts towards enhancing methodological rigour in future research. Where it is possible and applicable, randomized control trial methodologies should be employed, thereby strengthening the credibility and reliability of the research outcomes within this domain. The sample size of the projects varied markedly, and they were generally conducted within a single hospital rather than across multiple centres. This could indicate that research in this field for such interventions might be in its early stages of development. Typically, the studies were conducted in either a tertiary or secondary care setting, with only a few studies concentrating on specific clinical areas exclusively, suggesting a more centralized rather than global approach.

The scoping review had certain strengths and limitations. Despite employing a multi-database search approach, the search strategy used a relatively narrow set of terms to pinpoint relevant studies. Our rationale was grounded in the expectation that studies would commonly incorporate the term ‘diagnostic stewardship’ within the searchable text. This decision may have influenced the review by potentially reducing the recall of relevant studies. Furthermore, due to the period of time which has passed since the search was conducted, it is possible, based on the pattern of publications per year, that additional papers in this field have been published. The process of study selection involved three distinct single reviewers. Nevertheless, we managed to attain elevated levels of agreement among these individual reviewers during the abstract and title screening process, demonstrating good inter-rater reliability. Likewise, the data extraction phase engaged three separate single reviewers, which, akin to the screening process, could potentially introduce errors due to lack of verification. This was a pragmatic decision due to lack of resources for this stage of the scoping review. During the data extraction process, the team engaged in discourse about any challenges encountered, seeking assistance from senior screeners for clarification when data items were unclear.

## Conclusion

The approach of diagnostic stewardship appears to be a recent development and is gaining popularity. Studies that define their intervention as adopting a diagnostic stewardship approach are predominantly from the USA. This observation may indicate the influence of regional policy and practice drivers promoting this approach. However, diagnostic stewardship seems to have deviated from its intended course, morphing into a rather rudimentary instrument wielded not to enhance but to constrict the scope of testing. This is reflected in the majority of studies focusing on test reduction rather than test enhancement. Despite the WHO’s advocacy for an ideology that promotes a more holistic approach to quality improvement, it may be more appropriate to consider alternative regional narratives when categorizing these types of quality improvement interventions.

## supplementary material

10.1099/jmm.0.001831 Uncited Supplementary Material 1.

## Abbreviations

AMR: antimicrobial resistance
CDI: clostridium difficile infection
COVID-19: Coronavirus disease
GLASS: Global Antimicrobial Resistance Surveillance System
SARS CoV-2: Severe acute respiratory syndrome coronavirus 2
WHO: World Health Organiszation
